# Right-Sizing Thromboprophylaxis in Medical Inpatients

**DOI:** 10.1001/jamanetworkopen.2026.11399

**Published:** 2026-05-01

**Authors:** Anna L. Parks, Margaret C. Fang

**Affiliations:** **Author Affiliations:** Division of Hematology & Hematologic Malignancies, Department of Medicine, University of Utah, Salt Lake City (Parks); Division of Hospital Medicine, University of California, San Francisco, San Francisco (Fang).

For decades, venous thromboembolism (VTE) prevention has been considered a success of hospital quality improvement efforts. Large health system implementation initiatives have demonstrated that standardized VTE risk assessment embedded in the electronic health record can increase appropriate prophylaxis rates and may reduce hospital-associated VTE.^1^ Pharmacologic thromboprophylaxis has also been incorporated into hospital performance metrics.^2^ Over time, however, a more nuanced picture has emerged. While thromboprophylaxis clearly reduces VTE risk, the absolute event rates in modern hospitalized populations are low, and bleeding complications remain clinically meaningful.^3^ As a result, the central question facing clinicians is no longer whether prophylaxis works but rather which patients derive sufficient benefit to justify treatment.

Marti et al^4^ present a comprehensive network meta-analysis of randomized clinical trials evaluating pharmacologic thromboprophylaxis in acutely ill medical inpatients. Across 22 trials that included 43 840 patients, pharmacologic prophylaxis reduced symptomatic and clinically relevant VTE, with relative risk reductions of 30% to 40%. Low-molecular-weight heparin (LMWH) was associated with the most favorable balance of effectiveness and safety, whereas unfractionated heparin and direct oral anticoagulants were associated with higher risks of major bleeding; these findings align with prior evidence for LMWH in hospitalized patients. At the same time, absolute VTE event rates were modest (the pooled risk of symptomatic VTE without prophylaxis was 1.7% at 90 days), and mortality was unaffected by prophylaxis strategies. Given this low absolute risk, the authors emphasize the importance of patient selection, as bleeding risk may outweigh this modest absolute benefit of thrombosis prevention for most medical inpatients.

The central message of the study by Marti et al^4^ is the need for right-sizing prophylaxis in the modern era. Relative risk reductions associated with anticoagulant prophylaxis are substantial, but the clinical impact of treatment depends critically on baseline risk. When the probability of symptomatic VTE is low, even effective preventive therapies translate into modest absolute risk reductions and large numbers needed to treat. In such settings, risks and burdens—including bleeding, bruising, heparin-induced thrombocytopenia, and pain—may offset these modest benefits.

A key nuance in interpreting the study conclusions is contrasting them with prior literature syntheses. A 2022 network meta-analysis by Eck et al,^5^ which included 44 trials and 90 095 patients, concluded that pharmacologic prophylaxis was associated with broad reduction in VTE in hospitalized patients. One explanation for these differing conclusions lies in the studies included. In contrast to Eck et al,^5^ the Marti et al^4^ meta-analysis excluded trials with higher-risk populations, such as those admitted to the intensive care unit or those with cancer, acute stroke, or myocardial infarction. As a result, the trials included in the Marti et al^4^ meta-analysis may reflect a more typical general medical inpatient population with lower baseline VTE risk. Because the relative reductions in VTE and bleeding risks were similar across these 2 analyses, the less robust net benefit observed in the Marti et al^4^ study likely reflects the lower baseline risk of VTE in the included patients.

Ironically, the success of hospital VTE prevention initiatives may contribute to this tension. Hospitals have invested substantial effort in increasing prophylaxis use through standardized protocols, electronic order sets, and feedback and audit.^1^ National performance measures further reinforced these efforts by incorporating VTE prophylaxis into hospital reporting and accreditation programs.^2^ While these initiatives have successfully increased prophylaxis rates, their role in patient outcomes has been less clear. Observational studies have suggested that general medical patients in hospitals with higher prophylaxis rates do not necessarily experience lower VTE rates.^6^ At the same time, contemporary observational data indicate that thromboprophylaxis is frequently misaligned with patient risk, with both overuse among low-risk patients and underuse among high-risk patients.^7^ Together, these findings suggest that prevention strategies focused on increasing prophylaxis rates (or documentation of rates) may not target the patients most likely to benefit.

Right-sizing prophylaxis will require improvements in both VTE risk assessment and strategy implementation. Several models, including Padua and IMPROVE scores, have been developed to estimate VTE risk in hospitalized patients. However, in a prospective, multicenter, head-to-head comparison of existing risk assessment models, all analyzed models had poor prognostic accuracy and discriminative performance for estimating hospital-acquired VTE risk.^8^ Even with accurate risk assessment, in many settings, the barrier may be the difficulty of integrating a model into clinical practice. When a risk assessment model was embedded within the electronic health record in a cluster-randomized trial, clinician uptake was limited, underscoring how workflow integration and usability may be as important as predictive performance.^9^ Future efforts to improve prophylaxis will therefore require accurate strategies to assess VTE risk among hospitalized patients or tools that combine thrombotic and bleeding risk into a single decision framework and innovative methodologies to enable seamless integration into clinical decision support.

Finally, it is worth recognizing that the definition of benefit in thromboprophylaxis extends beyond clinical trial end points. For many patients, daily injections, bruising, and the psychological burden of anticoagulation are not trivial considerations. As with many preventive interventions in modern medicine, shared decision-making—particularly in patients at intermediate risk—may become increasingly important.

Pharmacologic prevention remains an important tool for reducing VTE in hospitalized medical patients, but its value depends on careful patient selection, thoughtful choice of agent, and implementation strategies that support clinicians in balancing thrombotic and bleeding risks. In the coming years, the success of hospital VTE prevention efforts may be judged not by how many patients receive prophylaxis but by how accurately health systems identify the patients who stand to benefit most.
